# Identification of the interaction of VP1 with GM130 which may implicate in the pathogenesis of CVB3-induced acute pancreatitis

**DOI:** 10.1038/srep13324

**Published:** 2015-08-28

**Authors:** Xiuzhen Li, Yanhua Xia, Shengping Huang, Fadi Liu, Ying Ying, Qiufang Xu, Xin Liu, Guili Jin, Christopher J. Papasian, Jack Chen, Mingui Fu, Xiaotian Huang

**Affiliations:** 1 Department of Medical Microbiology, School of Medicine, Nanchang University, Nanchang, Jiangxi, China; 2 Department of Basic Medical Science, School of Medicine, University of Missouri Kansas City, Kansas City, MO, USA; 3 Children’s Hospital of Jiangxi Province, Nanchang, Jiangxi, China; 4 Department of Pathophysiology, School of Medicine, Nanchang University, Nanchang, Jiangxi, China; 5 Shanghai Qingpu Center for Disease Control and Prevention, Shanghai, China; 6 The affiliated hospital of Jiangxi University of Traditional Chinese Medicine, Nanchang, Jiangxi, China; 7 Department of Biology and Wildlife, University of Alaska Fairbanks, Fairbanks, AK, USA

## Abstract

Coxsackievirus B3 (CVB3) is a causative agent of viral myocarditis, pancreatitis, and meningitis in humans. Although the susceptibility of CVB3-induced acute pancreatitis is age-dependent, the underlying mechanisms remain unclear. Here we identified the host factor Golgi matrix protein 130 (GM130) as a novel target of CVB3 during CVB3-induced acute pancreatitis. The viral protein VP1 interacted with GM130, disrupted GM130-GRASP65 complexes, and caused GM130 degradation, which may lead to disruption of the Golgi ribbon and development of acute pancreatitis in mice. Interestingly, the expression level of GM130 in mouse pancreas was age-dependent, which was nicely correlated with the age-associated susceptibility of CVB3-induced acute pancreatitis. Furthermore, interference RNA-mediated knockdown of GM130 significantly reduced CVB3 replication in HeLa cells. Taken together, the study identified GM130 as a novel target of CVB3, which may implicate in the pathogenesis of CVB3-induced acute pancreatitis.

Viral tropisms for specific organs have often been attributed to differential expression of cell surface receptors on those organs. For example, cell surface receptors for Coxsackie virus and adenovirus (CAR) have been previously implicated as important determinants for tissue tropisms for these viruses^1^. However, CAR mRNA expression in certain mouse organs did not correlate well with the susceptibility of the respective tissues, suggesting that other factors, including intracellular proteins may contribute to the regulation of viral infectivity through interaction with viral RNA or proteins^2^.

Human coxsackievirus B3 (CVB3) causes a variety of clinical manifestations in humans including myocarditis, pancreatitis, and meningitis^3^,^4^,^5^. Evidence from both clinical and animal studies has shown that the heart appears to become more resistant to CVB3 infection with increasing age^6^,^7^. In contrast, the pancreas is more susceptible to CVB3 infection with increasing age^8^,^9^,^10^. The mechanisms underlying variations in organ-specific tropisms with age are poorly understood. Moreover, despite the accumulation of data from experimental animal studies and molecular analyses, the pathogenic mechanisms by which CVB3 causes acute pancreatitis are unclear. CVB3 encodes four capsid proteins, VP1 through VP4. VP1, the most external and immunodominant capsid protein, binds to decay-accelerating factor (DAF) on the cell surface to initiate attachment, and also binds to CAR which functions as an internalization receptor, enabling CVB to initiate a productive infectious cycle. Many viral structural proteins, in addition to their structural function, contribute to cellular cytotoxic effects^11^,^12^,^13^,^14^. The role of VP1 protein in CVB-induced pathogenesis, however, is poorly understood.

Several types of viruses, including CVB, have been reported to target and disrupt the Golgi network, which disrupts host protein trafficking and antigen presentation on major histocompatibility molecules, thus impeding production of inflammatory cytokines, and recognition and destruction by cytotoxic T cells^15^,^16^,^17^,^18^,^19^,^20^,^21^,^22^,^23^. The molecular determinants that mediate viral disruption of Golgi functions, however, have not been well-characterized. In the present study, using a yeast two-hybrid assay (Y2H), we have identified GM130, a Golgi matrix protein, as a novel protein that interacts with the VP1 protein of CVB3. In an experimental mouse model of CVB3-induced acute pancreatitis, we observed that VP1 protein interacts with GM130 and causes GM130 degradation, leading to disruption of the Golgi ribbon and development of acute pancreatitis in adult mice. Interestingly, the expression pattern of GM130 in mouse tissues is tightly correlated with CVB3-induced pathogenesis. Further studies suggest that cellular expression of GM130 is required for CVB3 replication. Results of the current study provide novel insights into the molecular pathogenesis of CVB3 infections which may ultimately lead to targeted therapy for these infections.

## Results

### CVB3 infection causes acute pancreatitis but not myocarditis in 4–6 weeks-old mice

**Figure 1:**
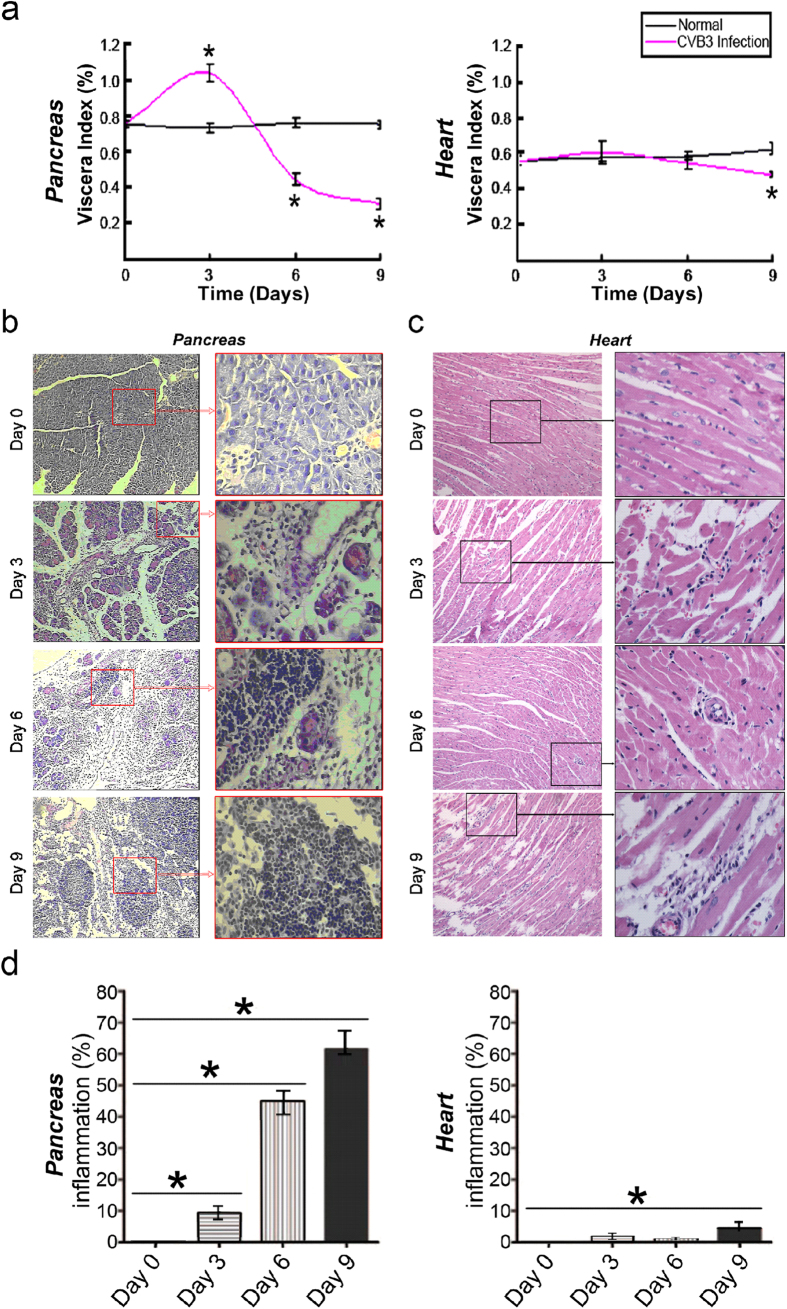
CVB3 infection causes acute pancreatitis but not myocarditis in adult mice. (**a**) Changes of visceral indexes of pancreas and heart. (left) Visceral index of the pancreas represent ratios of pancreas weight to body weight. (right) Visceral index of the heart represent ratios of heart weight to body weight. Data represent means ± s.d.; *P < 0.01 and n = 10 per group. (**b**) Serial sections from pancreas at day 0, 3, 6, and 9 p.i. with CVB3, stained with hematoxylin and eosin. Original magnifications, ×100 (left) or ×400 (right). (**c**) Serial sections from heart at day 0, 3, 6, and 9 p.i. with CVB3, stained with hematoxylin and eosin. Original magnifications, ×100 (left) or ×400 (right). (**d**) The extent of inflammatory changes in the pancreas and heart was quantified by measuring the percentage of tissue sections substantially infiltrated with inflammatory cells (Fig. 1d). Individual experiments were conducted 3 times with similar results. Data represent means ± s.d.; *P < 0.01 and n = 5 per group.

Human CVB3 infection can be mimicked in mice using a human pathogenic CVB3 strain^24^,^25^. CVB3 selectively infects several organs and causes different diseases such as myocarditis, pancreatitis, and meningitis. The organ tropism of CVB3 infection is dependent on age. For example, the heart becomes more resistant to CVB3 infection with increasing age^4^,^5^. In contrast, susceptibility to CVB3-induced acute pancreatitis increases with age^6^,^7^,^8^. To further confirm these observations, BALB/c mice at the age of 4–6 weeks were infected with CVB3. As expected, when uninfected control mice and mice infected with CVB3 were compared, significant differences were observed in visceral pancreatic indexes, calculated as the ratio of pancreatic mass to total body mass^26^. In contrast, no significant differences were observed in visceral heart indexes, when uninfected control mice and mice infected with CVB3 were compared (Fig. 1a). To further explore the pathologic effects of CVB3 on pancreas and heart, histologic examination of the pancreas and heart was performed at 0, 3, 6 and 9 days after infection. Inflammatory changes were detected in the pancreas at day 3, 6, and 9 p.i., but were non-existent to minimal in the heart (Fig. 1b,c). The extent of the inflammatory changes in the pancreas and heart was quantified by measuring the percentage of tissue sections substantially infiltrated with inflammatory cells (Fig. 1d).

### The VP1 protein of CVB3 interacts with GM130

**Figure 2:**
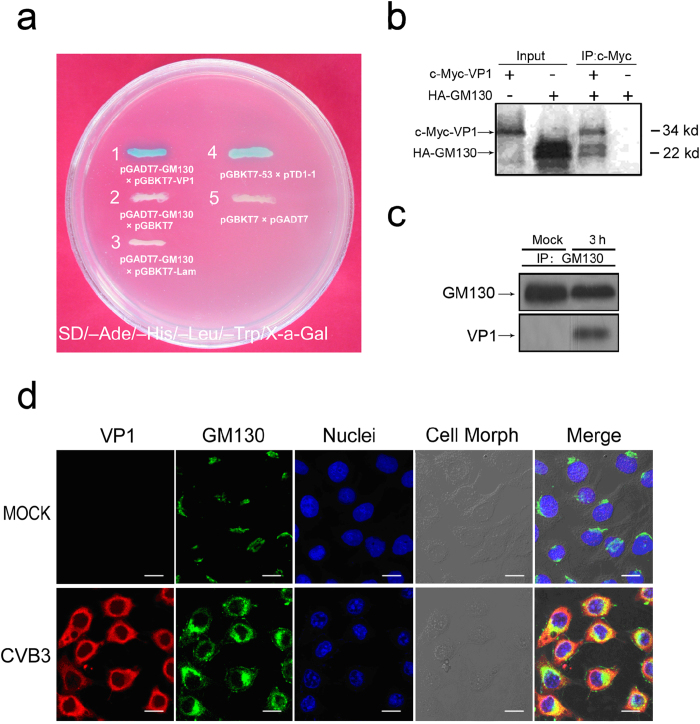
The interaction of CVB3 VP1 with human GM130. (**a**) CVB3 VP1-human GM130 interaction in an Y2H system. Yeast reporter strain AH109 was cotransformed with pGADT7-GM130 and pGBKT7-VP1, or with pGADT7-GM130 and pGBKT7, or with pGADT7-GM130 and pGBKT7-Lam, or with pGBKT7-53 and pTD1-1 (positive control), or with pGBKT7 and pGADT7 (negative control). The transformed cells were analyzed for α-Galactosidase activity. (**b**) *In vitro* protein-protein binding assay between CVB3 VP1 and human GM130. Tagged proteins of c-Myc-VP1 and HA-GM130 (199 amino acids of C-terminal) were translated *in vitro* with [^35^S] methionine incorporation. After translation, c-Myc-VP1 and HA-GM130 (199 amino acids of C-terminal) were immunoprecipitated with monoclonal anti-c-Myc antibody, and the antibody–protein complexes were resolved in 10% SDS–PAGE and subjected to autoradiography. (**c**) CVB3 VP1 formed a complex with GM130 *in vivo*. Lysates of CVB3-infected HeLa cells were immunoprecipitated with monoclonal anti-GM130 antibody. Immune complexes (Lane 2), the GM130 control in normal HeLa cells (Lane 1), and the VP1 control in lysates from CVB3-infected HeLa cells (Lane 3) were detected by western blot analysis using anti-GM130 antibody or anti-VP1 antibody. (**d**) Colocalization of CVB3 VP1 and human GM130 under a FLUOVIEW FV1000 confocal laser scanning microscope (Olympus). CVB3-infected HeLa cells at 3 h p.i. were subjected to double immunofluorescent staining with antibodies against VP1 (red) or GM130 (green). Co-localization of VP1 and GM130 is indicated in yellow in the merged images. Nuclear staining with DAPI is shown in blue. Scale bar, 10 μm.

Although it is well accepted that CVB3 infection causes pancreatitis, the pathogenic mechanisms leading to tissue damage remain unclear. Two general mechanisms that have been considered include direct viral cytopathic effects and host immune mediated tissue damage. We postulated that interactions between viral proteins and host proteins may contribute to the progress of pancreatitis during the early phase of viral infection, before the host’s adaptive immune response would be likely to produce any damage. To identify potential human proteins that interact with CVB3 proteins, we performed a yeast two-hybrid assay (Y2H)^27^. Plasmids were constructed by fusing DNA for CVB3 VP1 protein to DNA for GAL4-DNA binding domain, and the resultant hybrid proteins, produced in transformed yeasts, were used as bait. Prey proteins from a human HeLa cDNA library were expressed as fusions to GAL4-DNA activation domain, and mixed with CVB3 VP1-GAL4 DNA binding domain hybrids. Positive clones were identified based on quantitative α-Gal assays, and the clone with the highest expression was isolated. After BLAST searching (Basic Local Alignment Search Tool), the positive clone was matched to GM130 with 100% identity of the amino acid sequence and 97% of the cDNA sequence. Despite 3% difference in the Poly A region of the cDNA sequence between the positive clone and known GM130, we registered the sequences of cDNA with NCBI GenBank (Accession number AY823636). As a result of this screen, GM130 was identified as a potential binding partner of VP1 (Fig. 2a).

To verify the result from Y2H screening, tagged proteins of c-Myc-VP1 and HA-GM130 (199 amino acids of C-terminal) were translated *in vitro* in the presence of [^35^S] methionine and *in vitro* protein-protein binding assays were then performed. HA-GM130 could be pulled down with c-Myc-VP1 in the presence of anti-c-Myc antibody, while no protein could be detected in the absence of c-Myc-VP1 (Fig. 2b), indicating that VP1 could directly bind GM130 *in vitro*. Next, we carried out *in vivo* co-immunoprecipitation assay to confirm these interactions in mammalian cells. HeLa cells infected with CVB3 (MOI of 5) at 3 h p.i. were harvested. Cell lysates were immunoprecipitated with anti-GM130 antibody, and then analyzed by Western blot using anti-VP1 antibody. We confirmed that VP1 was associated with endogenous GM130 in CVB3-infected HeLa cells (Fig. 2c). These results suggest that VP1 interacts with GM130 *in vivo*.

We then examined the co-localization of VP1 and GM130 in cells. HeLa cells were infected with CVB3 (MOI of 5) for 3 h. The cells were processed with double immunofluorescent staining and visualized with a laser-scanning confocal microscope. Though VP1 was broadly distributed in the cytoplasm, there was at least partial co-localization of VP1 protein with endogenous GM130 in CVB3 infected HeLa cells (Fig. 2d). Taken together, our results show that VP1 interacts with GM130 in CVB3-infected HeLa cells.

### Specificity of VP1 interactions with GM130

**Figure 3:**
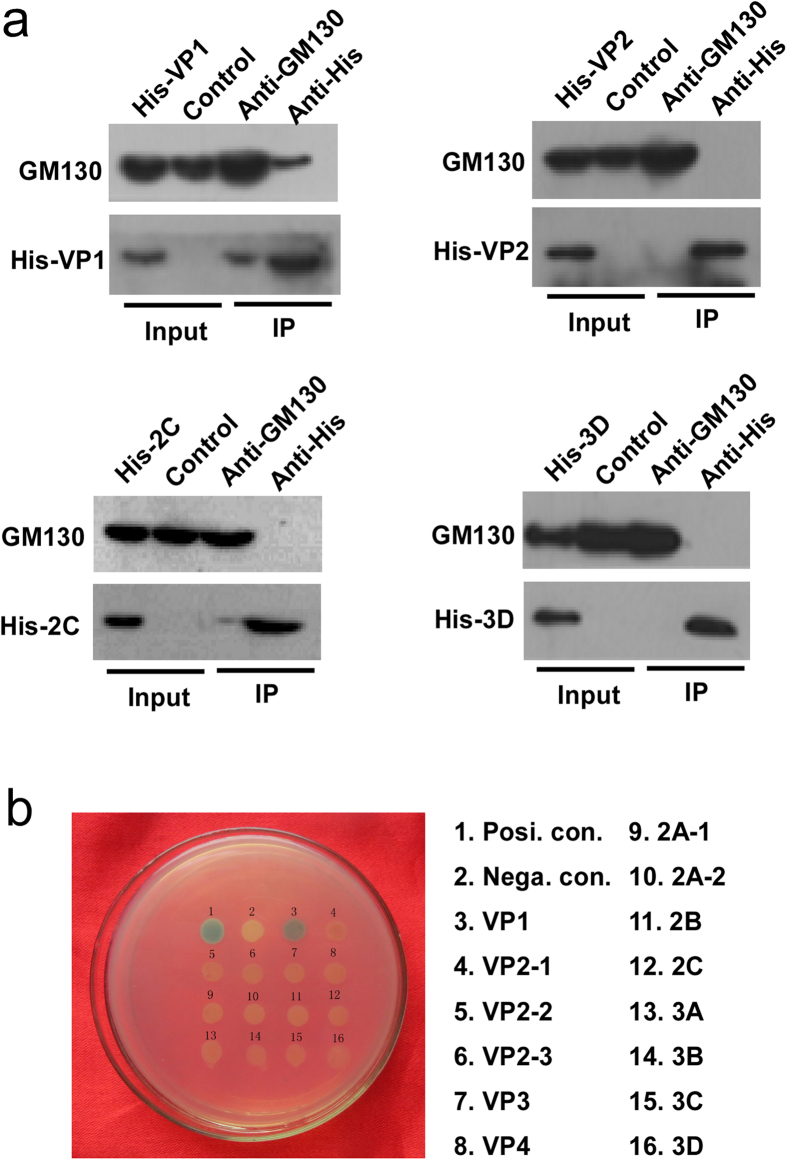
GM130 specifically interacts with VP1 of CVB3. (**a**) HeLa cells were transiently transfected with His-tagged VP-1, VP-2, 2C or 3D plasmids. The cell lysates were harvested and immunoprecipitated with monoclonal anti-GM130 or anti-His antibodies. Immune complexes and the input were analyzed by western blot analysis using anti-GM130 antibody or anti-His antibody. (**b**) Yeast reporter strain AH109 was cotransformed with pGADT7-GM130 and the different viral proteins in pGBKT7 as indicated. pGBKT7-53 served as a positive control, whereas pGBKT7 served as a negative control. The transformed cells were analyzed for α-Galactosidase staining.

To determine if GM130 specifically interacted with VP1, but not with other viral proteins, we transiently transfected His-tagged VP1, VP2, 2C or 3D plasmids into HeLa cells. After 24 hours, the cell lysates were harvested and co-immunoprecipitation was performed using anti-GM130 and anti-His. As shown in Fig. 3a, GM130 can be easily detected in anti-His-VP1 immunoprecipitates, and His-tagged VP1 can also be detected in anti-GM130 immunoprecipitates. The other viral proteins VP2, 2C and 3D were not associated with GM130. These results suggest that GM130 is a specific target of VP1. To further confirm these results, we utilized the Y2H approach. Briefly, a series of viral proteins in pGBKT7 vector were co-transformed with pGADT7-GM130 vector into yeast cells. Yeast transformants positive for prey-bait interaction were selected on plates lacking adenine, histidine, leucine, and tryptophan with X-α-Gal and assayed for α-galactosidase activity. As shown in Fig. 3b, only VP1 showed a positive interaction with GM130.

### Interacting domains within CVB3 VP1 and GM130

**Figure 4:**
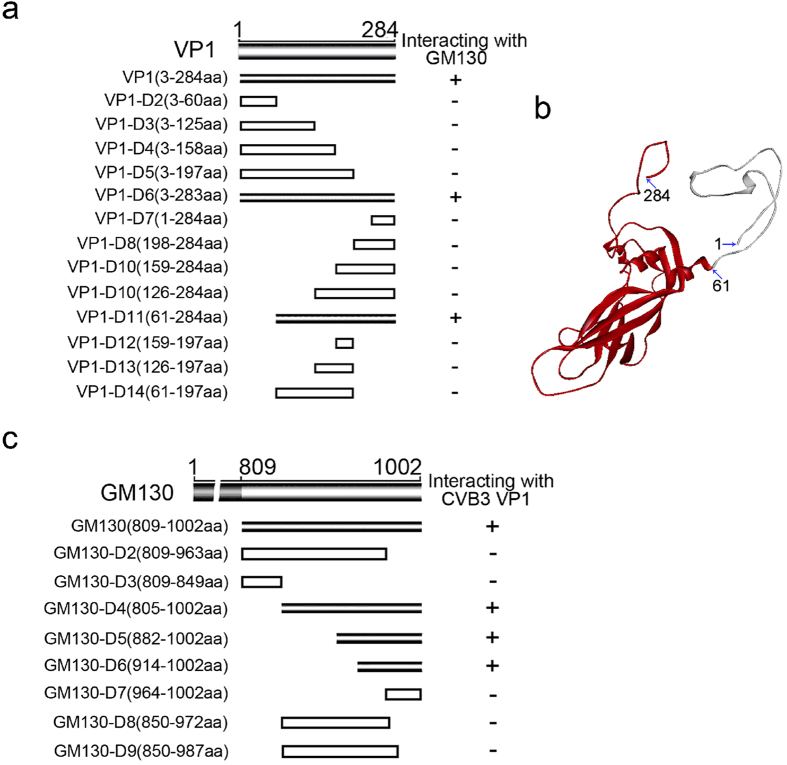
Mapping the regions of VP1 and GM130 that are required for their interaction. (**a**) The regions of VP1 interacting with GM130 were mapped by a Y2H assay. Truncation/deletion mutants of VP1 in pGBKT7 vector were used as the bait plasmids. These were tested for interaction with GM130 prey cloned into pGADT7 vector by α-Galactosidase assays performed on yeast colonies cotransformed with the prey and bait plasmids, and selected on plates lacking adenine, histidine, leucine, and tryptophan with X-α-Gal. “+” or gray sticks indicate VP1 prey constructs that interacted with GM130, while “–” or hollow bars indicate constructs that did not interact with GM130. (**b**) Molecular Modeling of CVB3 VP1 protein (GenBank accession number: M88483) on the basis of the published crystal structure of CVB3, as predicted by SWISS-Model server and Discovery Studio Visualizer software. The region of VP1 that binds to GM130 is colored in red. (**c**) The regions of GM130 interacting with VP1 were mapped by an Y2H assay. The prey proteins, the truncation/deletion mutants of GM130, were tested against the bait VP1. Shown are the results of Y2H α-Galactosidase assays performed on yeast colonies cotransformed with the prey and bait plasmids and selected on plates lacking adenine, histidine, leucine, and tryptophan with X-α-Gal. “+” or gray sticks indicate GM130 prey constructs that interacted with VP1, while “–” or hollow bars indicate constructs that did not interact with VP1.

To further characterize the interaction between the two proteins, we attempted to map the regions of VP1 that interacted with GM130 utilizing the Y2H approach. A series of VP1 truncation/deletion mutants in pGBKT7 vector were co-transformed with pGADT7-GM130 vector into yeast cells. Yeast transformants positive for prey-bait interactions were selected on plates lacking adenine, histidine, leucine, and tryptophan with X-α-Gal and assayed for α-galactosidase activity. The VP1 truncation segment spanning amino acids 61–284 interacted with GM130 while segments spanning amino acids 3–197 and 126–284 failed to interact with GM130 (Fig. 4a,b).

We next mapped regions of GM130 interacting with VP1. GM130, a 1002 amino acid protein, is an extended rod-like protein with six coiled-coil domains in the middle region. Mapping was also performed by utilizing Y2H assays, wherein the prey (GM130 truncation/deletion segments in pGADT7 vector) and the bait (pGBKT7-VP1 vector) were co-transformed into yeast cells. Yeast transformants positive for prey-bait interactions were selected as above and assayed for α-galactosidase activity. The GM130 truncation segments spanning amino acids 809–963, 850–987 and 964–1002 failed to interact with VP1 (Fig. 4c). However, the segment spanning amino acids 914–1002 interacted with VP1. These results suggested that VP1 specifically interacted with the Carboxyl-terminus (amino acids 914–1002) of GM130. Interestingly, the last 100 amino acids of GM130, including the free C-terminus, have been previously reported to contain the binding sites for a Golgi apparatus-associated protein, GRASP65^28^, suggesting that the interaction between CVB3 VP1 and GM130 may interfere with formation of GM130-GRASP65 complexes.

### CVB3 infection dissociates GM130-GRASP65 complexes

**Figure 5:**
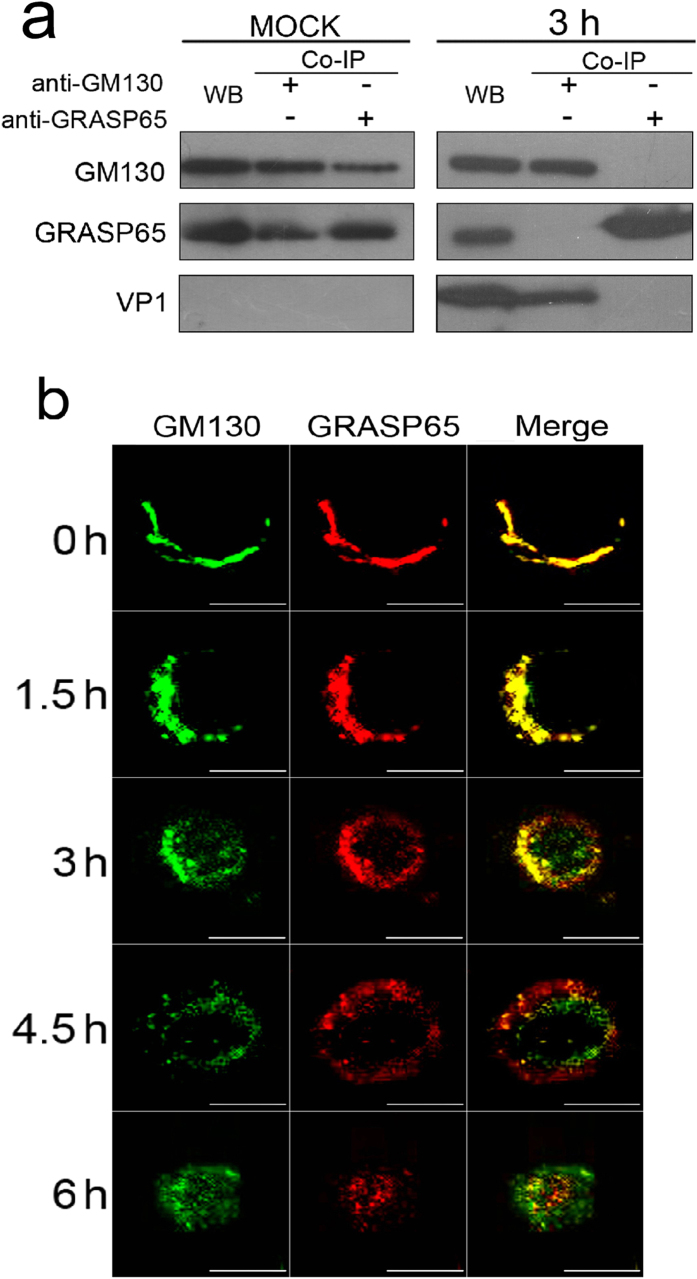
CVB3 infection dissociates GM130-GRASP65 complexes in HeLa cells. (**a**) Cellular lysates of HeLa cells with or without CVB3 infection (MOI of 5) were immunoprecipitated with antibodies against GM130 or GRASP65. Immunoprecipitates and cellular lysates were analyzed using antibodies against GM130, VP1 or GRASP65 by western blot. (**b**) Immunofluorescent staining with antibodies against GM130 or GRASP65 at indicated time points in HeLa cells infected with CVB3 (MOI of 5). Scale bars, 10 μm.

The Golgi apparatus of most mammalian cells is a single-copy organelle and forms a continuous ribbon of interconnected stacks of flat cisternae. The formation of the Golgi ribbon requires interactions between the Golgi matrix proteins GM130 and GRASP65 during Golgi assembly^29^. Since the results above suggest that VP1 binds to sites on GM130 critical to the formation of GM130-GRASP65 complexes, we investigated whether VP1 interaction with GM130 could dissociate GM130-GRASP65 complexes in CVB3-infected HeLa cells. First, we confirmed the presence of GM130-GRASP65 complexes in HeLa cells after mock infection with PBS by immunoprecipitation assay with antibodies against GM130 or GRASP65 (Fig. 5a). Next, we examined total cellular lysates of Hela cells 3 h after infection with CVB3 by immunoprecipitation with antibodies against GM130 or GRASP65. Immunoprecipitates and cellular lysates were subjected to western blot analysis using antibodies against GM130, VP1 or GRASP65. Figure 5a showed that expression of VP1 interacted with GM130, which interfered the interaction of GRASP65 with GM130. These results indicate that VP1 may directly dissociate GM130-GRASP65 complexes by binding to GM130. These results were further supported by confocal microscopy (Fig. 5b). Although GM130-GRASP65 clearly co-localized in the Golgi of HeLa cells at 0 h p.i. with CVB3, GM130 and GRASP65 tended to separate from one another in CVB3-infected HeLa cells beginning 3 h p.i,; by 6 h.p.i, the distribution of these two proteins had changed substantially. (Fig. 5b). Collectively, these results indicate that direct interactions between CVB3 VP1 and GM130 can dissociate GM130-GRASP65 complexes.

### CVB3 infection promotes GM130 degradation and disrupts Golgi ribbons in HeLa cells

**Figure 6:**
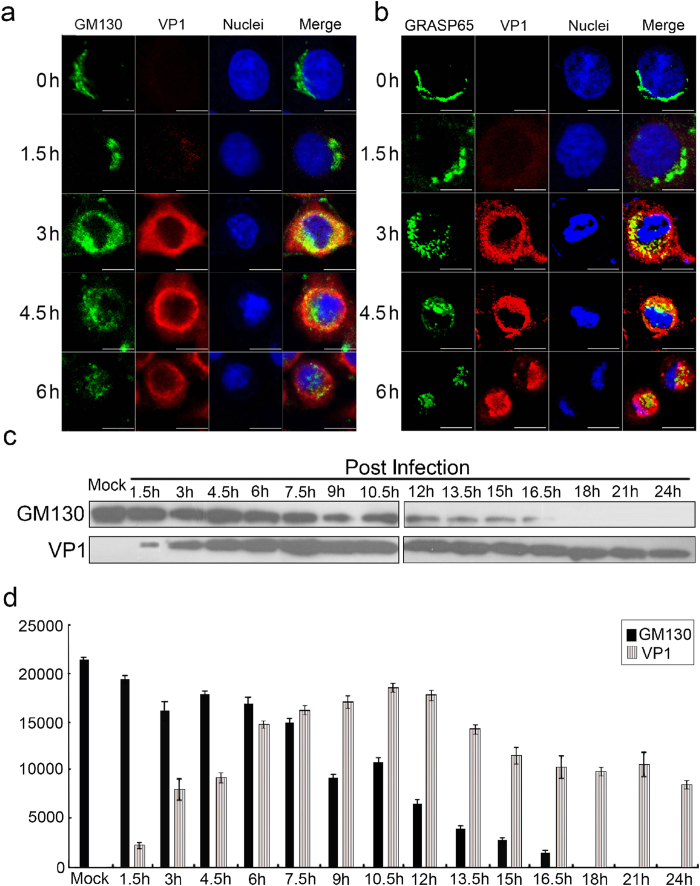
CVB3 infection promotes GM130 degradation and disrupts Golgi ribbons in HeLa cells. (**a,b**) Immunofluorescent staining with antibodies against GM130, VP1 or GRASP65 at indicated time points in Hela cells infected with CVB3 (MOI of 5), along with staining of nuclei (DAPI). Scale bars, 10 μm. (**c**) The expression of GM130 and VP1, as determined by western blot, at indicated time points in HeLa cells infected with CVB3 (MOI of 5). (**d**) The abundance of GM130 and VP1, as examined by Western blot, in CVB3-infected HeLa cells. Data represent means ± s.d. These experiments were repeated three times.

The Golgi apparatus is composed of multiple stacks of polarized cisternal membranes that are held in a juxtanuclear position by microtubules. These cisternal membranes are aligned laterally in a ribbon-like structure, called the Golgi ribbon, which is unique to mammalian cells. In the HeLa cells infected with CVB3, the organization of the Golgi apparatus (marked by GM130 and GRASP65) changed markedly over time. At 0–1.5 h.p.i. GM130 and GRASP65 are found in ribbon like structures adjacent to the nucleus. After 3 h.p.i., however, this organization changes markedly and both proteins become progressively dispersed, in a time-dependent manner, as discrete small elements in the perinuclear region (Fig. 6a,b). An apparent decrease of these small elements of the Golgi was also observed in CVB3-infected HeLa cells from 3 h to 6 h p.i. Concomitantly, the level of GM130 protein progressively decreased to undetectable levels at 18 h p.i. in CVB3-infected HeLa cells (Fig. 6d,c). Taken together, these results suggest that CVB3 infection promotes GM130 degradation and Golgi ribbon disruption, which may contribute to the pathogenesis of CVB3-induced acute pancreatitis.

### Overexpression of VP1 also promotes GM130 degradation and disrupts Golgi ribbons in HeLa cells

**Figure 7:**
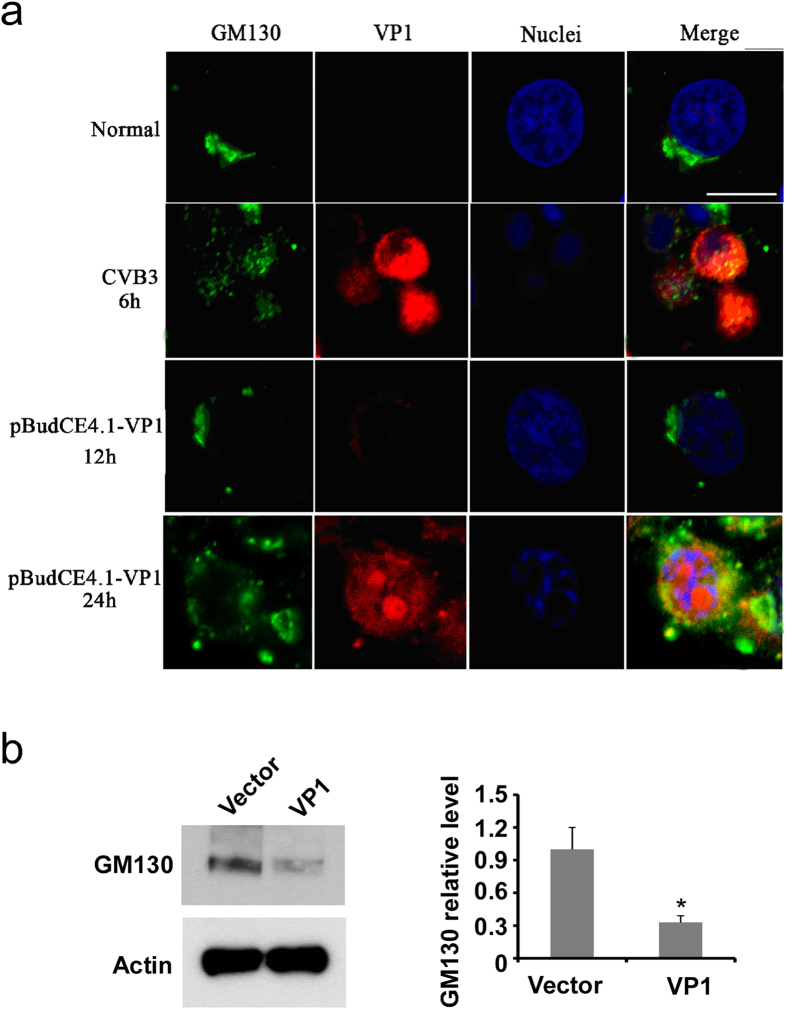
Overexpression of VP1 also promotes GM130 degradation and disrupts Golgi ribbons in HeLa cells. (**a**) HeLa cells were transfected with VP1 plasmids or infected with or without CVB3 for varied times as indicated. GM130 and VP1 were detected by immunofluorecent staining and visualized by confocal microscopy. Bar = 10 μm. (**b**) HeLa cells were transfected with empty vector or VP plasmid for 24 hours. The expression of GM130 in the cell lysates was examined by Western blot with GM130 antibody. Actin was also probed to serve as a loading control. The relative GM130 protein level was analyzed and shown on the right of the blots. *P < 0.01 vs vector group.

**Figure 8:**
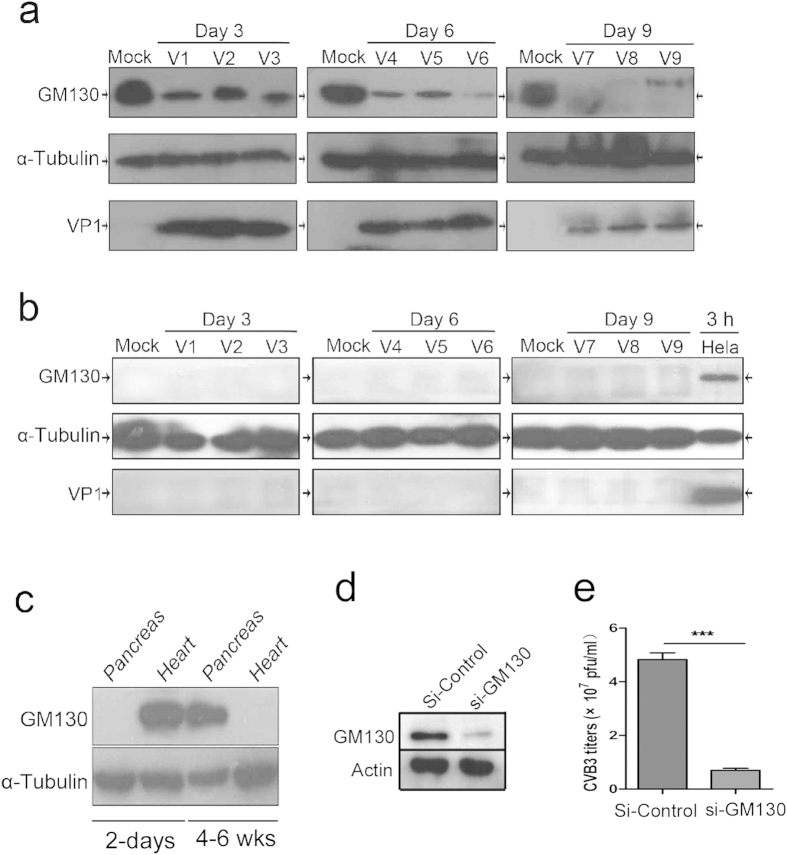
GM130 expression may contribute to the tissue specificity of CVB3. (**a**) GM130 expression in the pancreas from the mice at the age of 4–6 weeks that were infected with CVB3. Tissue proteins were collected at the indicated time points and analyzed by Western blot with anti-GM130, anti-VP1 and anti-α-Tubulin. (**b**) GM130 expression in the hearts from the mice at the age of 4–6 weeks that were infected with CVB3. Tissue proteins were collected at the indicated time points and analyzed by Western blot with anti-GM130, anti-VP1 and anti-α-Tubulin. (**c**) GM130 expression in the pancreas and hearts from the mice at the age of 2 days or 4–6 weeks was examined by Western blot. Tubulin was probed to serve as a loading control. (**d**) Generation of siRNA-mediated knockdown stable cell lines. HeLa cells were transfected with the plasmids for si-Control or si-GM130. Positive clones were selected by incubation with G418. The knockdown stable cell lines were analyzed by western blot analysis with anti-GM130. (**e**) CVB3 replication in GM130-knocking down stable cell lines.

To determine whether VP1 protein triggers GM130 degradation and Golgi ribbon disruption during CVB3 infection, we transfected HeLa cells with an expression vector encoding VP1. As shown in Fig. 7a, overexpression of VP1 is sufficient to induce the disruption of the Golgi ribbon, similar to that observed with CVB3 infection. Moreover, overexpression of VP1 promoted GM130 degradation (Fig. 8b). These results suggest that VP1 protein interacts with GM130 and promotes GM130 degradation and Golgi ribbon disruption during CVB3 infection. This appears to represent a novel mechanism by which CVB3 infection could contribute to the pathogenesis of pancreatitis.

### The expression of GM130 is correlated with CVB3 replication and pathogenesis of CVB3-induced acute pancreatitis

GM130 was initially identified as a *cis*-Golgi matrix protein, but further studies have shown that it also mediates trafficking between the ER and Golgi apparatus^30^,^31^. To determine if the expression of GM130 is correlated with CVB3 replication and pathogenesis, we examined the expression of GM130 and VP1 proteins in the mouse heart and pancreas at the age of 4–6 weeks that were infected with CVB3. As shown in Fig. 8a, GM130 was highly expressed in the mouse pancreas. VP1 protein was also detected in pancreases from virus-infected mice. Similar to the results in HeLa cells, CVB3 infection caused GM130 protein degradation in the pancreas. In contrast, GM130 was not expressed in the mouse hearts (Fig. 8b), and VP1 was not detected in CVB3-infected mouse hearts. These results suggest that GM130 mediates the interaction with CVB3 and contributes to the infectivity of CVB3. To further explore these findings, we examined the expression of GM130 in heart and pancreas from BALB/c mice at the age of 2 days or 4–6 weeks by Western blot analysis. Interestingly, GM130 was highly expressed in the pancreas but not in the heart from the mice at the age of 4–6 weeks. In contrast, GM130 is abundant in the heart but not expressed in pancreas from the mice at the age of 2 days (Fig. 8c). This expression pattern is tightly correlated with the age-associated susceptibility of heart and pancreas to CVB3 infection in mice (Fig. 1). To further determine if GM130 expression is required for CVB3 replication, we used siRNA interference to knockdown the expression of GM130 in HeLa cells (Fig. 8d). These cells were subsequently infected with CVB3. As shown in Fig. 8e, CVB3 titers were significantly decreased in the si-GM130 knockdown HeLa cells. Taken together, these results strongly suggest that the interaction of GM130 with VP1 protein may be essential for replication of CVB3 and for the CVB3-induced pathogenesis of myocarditis and pancreatitis.

## Discussion

It has been widely reported that many RNA viruses, including CVB3, target the endoplasmic reticulum and Golgi network^32^,^33^,^34^,^35^,^36^,^37^. Through this mechanism, the viruses remodel intracellular membranes to generate specialized sites for RNA replication. Additionally, the viruses suppress the immune response by destroying the ER-to-Golgi protein secretion pathway, which affects the production of inflammatory cytokines such as interleukin 6 (IL-6), IL-1 and IL-13, and loading of viral peptides onto MHC class I molecules for recognition by cytotoxic T cells. Although direct targeting of the Golgi network has been implicated in the pathogenesis of viral infections, little information exists regarding the molecular determinants that mediate this targeting. The present study identified a Golgi matrix protein, GM130, as a novel target of CVB3 VP1. The interaction of VP1 with GM130 disrupts GM130-GRASP65 complexes, leading to GM130 degradation, and Golgi disruption. This certainly appears to be a potentially important molecular mechanism that could contribute to the pathogenesis of CVB3-induced acute pancreatitis.

It is well known that CVB3 has the potential to infect multiple human organs, resulting in several distinct clinical manifestations such as myocarditis, pancreatitis, and meningitis^1^,^2^,^3^. The susceptibility of CVB-induced pancreatitis or myocarditis is age-dependent^6^,^7^,^8^. The underlying mechanism for these distinct clinical manifestations in different age groups has not been well explained. Importantly, we observed that GM130 expression in HeLa cells was required for CVB3 replication. Consequently, it is quite interesting that we found that the level of GM130 expression in the heart and pancreas is dependent on age. GM130 expression in mouse pancreas is increased with increasing age, whereas the expression level of GM130 in mouse heart is decreased with increasing age. This corresponds directly with age associated tissue tropisms of CVB3 viral infections in mice. Consequently, results from the present study provide new insights into the age associated tissue tropisms of CVB3 infections.

Previous studies from many groups have demonstrated that many other viral proteins of CVB such as 3A and 2B targeted the Golgi, causing Golgi disruption which disrupted intracellular protein trafficking^38^,^39^. Moreover, Sasaki *et al.* observed that Aichi viral proteins 2B, 2BC, 2C, 3A and 3AB interacted with a Golgi protein, acyl-coenzyme A binding domain containing 3 (ACBD3). ACBD3 further recruited phosphatidylinositol 4-kinase IIIβ (PI4KB) to form a protein complex, which is essential in viral RNA replication^40^. In the present study, we observed that GM130 specifically interacted with the viral protein VP1, but not the other viral proteins of CVB3, which suggests that distinct proteins from different viruses may target different Golgi proteins to disrupt Golgi function and promote viral pathogenesis.

The Golgi apparatus of most mammalian cells is a single-copy organelle that forms a continuous ribbon of interconnected stacks of flat cisternae. The formation of the Golgi ribbon requires interaction between the Golgi matrix proteins GM130 and GRASP65 during Golgi assembly. Through detailed mapping of interacting domains, we found that VP1 of CVB3 binds to the C-terminus region (amino acids 914–1002) of GM130, which is also a binding site for the Golgi apparatus-associated protein GRASP65. Further studies demonstrated that the direct interaction of VP1 with GM130 dissociated GM130-GRASP65 complexes, a mechanism that appears to explain disruption of Golgi ribbon during CVB3 infections.

We also showed that CVB3 infection caused GM130 protein degradation both in HeLa cells and in pancreases from adult mice. Moreover, transfection of VP1-encoding plasmid into HeLa cells also triggered GM130 degradation, suggesting that VP1 binding to GM130 may recruit E3 ligase to the protein complex. This would be anticipated to promote GM130 ubiquitination and degradation, which may also contribute to the pathogenesis of CVB3-mediated acute pancreatitis. Further investigation is necessary to decipher the detailed molecular mechanisms by which VP1 induces GM130 degradation.

The incidence of CVB3-mediated myocarditis is deceased with increasing age^4^,^5^. Further investigation is needed to determine whether the interaction of VP1 with GM130 also contributes to the pathogenesis of CVB3- induced myocarditis. In addition, VP1 is a highly conserved viral protein within the Coxsackievirus viruses, with more than 70% identity among subgroup VB1, CVB3–5, and CVA9^41^,^42^,^43^. It is possible that VP1 homologs of those serotypes may also have the capacity to bind GM130, thereby contributing to the pathogenesis of infections with other Coxsackie viruses. Further understanding of the role of GM130 in the pathogenesis of CVB3 infections, as well as infections with similar serotypes has significant potential for the development of a new generation of antiviral drugs.

## Methods

### Ethics Statement

HeLa cells were purchased from ATCC. The animal protocol in this study was approved by Nanchang University Animal Care and Use Committee (NCU1239). All animal experiments were performed according to the guidelines for the Care and Use of Laboratory Animals (Ministry of Health, PR China, 1998) and the guidelines of the Laboratory Animal Ethical Commission of Nanchang University.

### Cell culture, virus, and mice

HeLa cells were grown and maintained in RPMI 1640 medium supplemented with 10% heat-inactivated fetal calf serum. CVB3 (Nancy strain) was propagated in HeLa cells and purified by a method previously described by Henke *et al.*^44^. Cells were infected with a Multiplicity of Infection (MOI) of 5 throughout the study. Newborn BALB/c (H-2^d^) mice (within 48 h after birth) and adult male mice (beyond 4 weeks of age), were obtained from Experimental Animal Centre of Nanchang University, housed and bred under pathogen-free conditions at BSL 2 level. For CVB3 infection, mice 4 and 6 weeks old were inoculated intraperitoneally with 1 × 10^5^ PFUs CVB3. Experimental groups consisted of a minimum of three mice, and experiments were repeated for three times

### Vector constructs

We engineered plasmids pGBKT7-VP1 and pGADT7-GM130 using standard molecular biology protocols. We generated recombinant plasmids used as mapping the regions in VP1 and GM130 that are required for their interaction for Y2H by cloning the different truncation/deletion mutants of CVB3 VP1 and GM130 in pGBKT7 vector (between the Nde I & Pst I sites) in frame with the vector’s GAL4 DNA Binding Domain (BD) and pGADT7 vector (between the EcoR I & BamH I sites) in frame with the vector’s GAL4 Activation Domain (AD). The deletion mutants of both VP1 and GM130 were generated by PCR with either pGBKT7-VP1 or pGADT7-GM130 as the templates respectively.

### Histopathology

Serial 4-μm sections were used for hematoxylin and eosin staining and immunohistochemistry. We randomly cut three paraffin-embedded sections of each pancreas and heart from mice in indicated groups. To evaluate the severity of pancreatitis or myocarditis, we stained them with hematoxylin and eosin.

### Indirect immunofluorescence labelling and confocal microscopy

We applied fluorescein-conjugated donkey anti-goat IgG (Chemicon), rhodamine-conjugated donkey anti-mouse IgG (Chemicon), or Cy5-conjugated goat anti-rabbit IgG (Abcam) after an overnight incubation of CVB3-infected Hela cells with primary antibodies. The primary antibodies used were mouse anti-human GM130 (BD Transduction Laboratories), goat anti-human GM130 (Santa Cruze), rabbit anti-human GRASP65 (Thermo Scientific) or mouse antienterovirus VP1 clone 5-D8/1 (Dako). We used DAPI for counterstaining. We analyzed images obtained with an FLUOVIEW FV1000 confocal laser scanning microscope (Olympus) with Olympus FV1000 software.

### Western blots

The proteins from tissues and cells were separated by standard 10% SDS-PAGE followed by transfer of the proteins to a PVDF membrane. The proteins were detected by the following primary antibodies: GM130 (BD Transduction Laboratories), VP1 (Dako) and α-tubulin (Abcam), and followed by incubation with a secondary sheep anti-mouse IgG horseradish peroxidase. Staining was performed with ECL western blot detection reagent (Millipore). Antibody to α-tubulin served as the endogenous control. All experiments were performed in triplicate.

### Yeast two-hybrid screen and assay

Matchmaker Gal4 two-hybrid system (Clontech, Palo Alto, CA) were used for yeast two-hybrid screening according to the manufacturer’s instructions. The bait construct consisted of CVB VP1 residues 3–284 cloned in frame into the plasmid pGBKT7. The yeast strain AH109 was cotransformed with the bait and a human Hela cDNA library (Clontech) as prey. We confirmed the initial interaction by co-transforming each unique prey plasmid into AH109 with the VP1 bait construct and screening each combination on high-stringency media in the absence of leucine, tryptophane, adenine and histidine and in the presence X-α-Gal. As negative controls, yeast cells were co-transformed with empty Gal4BD vector pGBKT7 and empty Gal4AD vector pGADT7, or with pGADT7-GM130 and pGBKT7, or with pGADT7-GM130 and pGBKT7-Lam (non-related protein). As a positive control, yeast cells were cotransformed with pGBKT7-53 and pTD1-1. We identified the regions in VP1 and GM130 that are required for their interaction by bait plasmids composed of recombinant pGBKT7 vectors coding the different truncation/deletion mutants of CVB3 VP1 and prey plasmids contained recombinant pGADT7 vectors coding the different truncation/deletion mutants of GM130 with Matchmaker system. The yeast strain AH109 was co-transformed with prey and bait plasmids using lithium acetate. Yeast transformants positive for prey-bait interaction were selected on plates lacking adenine, leucine, tryptophan and histidine but containing X-α-Gal.

### *In vitro* protein-protein binding assay

We performed a TNT quick-coupled *in vitro* transcription-translation system (Promega) to obtain proteins used in protein-protein binding assays according to the manufacturer’s procedures. ^35^S-Methionine (Amersham Pharmacia Biotech) was included in the reaction for the purpose of labeling the synthesized proteins. The templates were pGBKT7-VP1 and pGADT7-GM130. The protocol of the *In Vitro* protein-protein binding Assay has been previously described^45^. ^35^S-Methionine-labeled HA-GM130 and c-Myc-VP1 were incubated with monoclonal anti-c-Myc antibody (Clontech) in lysis buffer (50 mM Tris–HCl [pH 7.5], 150 mM NaCl, 1 mM EDTA, 0.1% NP-40, 1 mM phenylmethylsulfonyl fluoride), followed by adsorption to BSA blocked protein A/G plus-agarose (Santa Cruz). The beads were washed thrice with lysis buffer (50 mM Tris–HCl [pH 7.5], 150 mM NaCl, 1 mM EDTA, 0.1% NP-40). The antibody–protein complexes were eluted and then resolved in 10% SDS–PAGE and subjected to autoradiography.

### Co-immunoprecipitation

We performed co-immunoprecipitation as described previously^46^. CVB3-infected Hela cells at 3 h p.i. were lysed with lysis buffer (150 mM NaCl, 20 mM Tris HCl, 0.1% NP-40, pH 7.4) and nuclei were removed by a 10-min low-speed centrifugation step. Precleared cell lysates were incubated with or without polyclonal antibodies (1:500) against GM130 (Santa Cruz Biotechnology) or irrelevant IgG for 1 h, and for an additional hour with Protein A/G Agarose beads (Santa Cruz Biotechnology) by gentle rotation at 4 °C. The beads were washed three times in lysis buffer with protease inhibitors, and two times in a wash buffer (100 mM Tris pH 7.4) with 500 mM LiCl before suspending in 1 ×SDS load buffer. The proteins were analyzed by immunoblot with specific antibodies against GM130, VP1 or GRASP65.

### Statistical analyses

All results are expressed as means ± s.e.m. Unpaired two-tailed Student’s t-tests were used to determine statistically significant differences for all comparisons by SPSS13.0 statistical package (SPSS).

## Additional Information

**How to cite this article**: Li, X. *et al.* Identification of the interaction of VP1 with GM130 which may implicate in the pathogenesis of CVB3-induced acute pancreatitis. *Sci. Rep.* **5**, 13324; doi: 10.1038/srep13324 (2015).
